# Marker-Assisted Selection of Soybean E-Locus Alleles Using Functional and Linked Markers: Phenotypic Validation Under High-Latitude Conditions

**DOI:** 10.3390/life16101663

**Published:** 2026-10-03

**Authors:** Bekzhan Maikotov, Gaziza Zhumaliyeva, Svetlana Didorenko, Rystay Kushanova, Alyona Zinchenko, Sholpan Bastaubayeva, Zaheer Ahmed, Raushan Yerzhebayeva

**Affiliations:** 1 Plant Biotechnology Laboratory, Kazakh Research Institute of Agriculture and Plant Growing, Almalybak 040909, Kazakhstan; maikotov.bekzhan@mail.ru (B.M.); gaziza_jumalieva@mail.ru (G.Z.); 2 Oilseed Crops Laboratory, Kazakh Research Institute of Agriculture and Plant Growing, Almalybak 040909, Kazakhstan; svetl_did@mail.ru (S.D.); kushanova60@mail.ru (R.K.); 3 Department of Agronomy, Breeding and Biotechnology, Kazakh National Agrarian Research University, Almaty 050010, Kazakhstan; 4 Oil Crops Department, Zarechnoye Experimental Station, Zarechnoye 111108, Kazakhstan; zinchenko.av@inbox.ru; 5 Department of Plant Breeding and Genetics, University of Agriculture, Faisalabad 38000, Pakistan; zaheer.ahmed@uaf.edu.pk

**Keywords:** soybean, marker-assisted selection, photoperiod response, *E* loci, early maturity, adaptation, introgression, high latitudes

## Abstract

Photoperiod sensitivity is one of the main factors limiting soybean adaptation to high-latitude conditions. The aim of this study was to perform marker-assisted selection (MAS) at the *E1*, *E3*, *E4*, and *E7* loci in three soybean F_2_ populations (F10, F13, and U4), followed by field evaluation of their F_3_ progenies in Northern Kazakhstan. Individual plants were genotyped with functional PCR-based markers for *E1*, *E3*, and *E4,* and with linked SSR markers for *E7*. This allowed us to select plants carrying target alleles and marker profiles. In total, 110 F_3_ progenies were tested in the Kostanay Region, and 80 of them (72.7%) reached full maturity. The phenotypic associations of the *E* loci and differed among genetic backgrounds. In F10, *e3-fs* was strongly associated with earlier flowering and completion of maturation. In F13, *e4-SORE-I* was associated with earlier flowering, a higher frequency of completed maturation, and earlier maturity. Also in F13, SSR marker classes associated with *E7* were significantly related to maturity status and days to maturity. Among the multilocus classes in F13, the target combination *e3-fs*/*e3-fs e4-SORE-I*/*e4-SORE-I* together with the SSR marker profile associated with *e7*/*e7*, showed the earliest mean flowering and maturation times. Based on the field results, 14 F_3_ progenies were selected for further breeding work. These progenies completed maturation within 90–110 days and met additional agronomic selection criteria. Hybridization combined with MAS enabled targeted selection of breeding material carrying the valuable alleles *e1-nl*, *e3-fs*, and *e4-SORE-I*, as well as favorable multilocus profiles. The results highlight the value of combining multilocus MAS with field evaluation in the target environment for developing early-maturing, photoperiod-adapted soybean breeding material for Northern Kazakhstan.

## 1. Introduction

Soybean (*Glycine max* (L.) Merr.) is one of the leading legume-oilseed crops, cultivated worldwide across a wide range of latitudes [1]. Expanding its cultivation area towards northern regions requires the development of varieties capable of completing a full development cycle under conditions of long summer photoperiod, a relatively short frost-free period, and specific temperature regimes [2,3]. One of the main biological factors limiting the geographic adaptation of soybean is photoperiod sensitivity, which determines the timing of the transition to flowering and subsequent maturation [3,4,5].

Soybean is a short-day plant characterized by considerable genotypic variability in photoperiod response [6]. Under high-latitude long-day (LD) conditions during the growing season, photoperiod-sensitive genotypes delay the transition to the flowering phase, which can extend the vegetative growth period and lead to later maturity [6,7]. Given the limited duration of the frost-free period, this increases the risk of incomplete plant maturation before the onset of unfavorable autumn temperatures and, consequently, reduces yield and seed quality [7]. Therefore, reducing photoperiod sensitivity and ensuring timely maturation are important breeding objectives for soybean in northern regions.

The photoperiod response and the time to flowering and maturity in soybean have a complex genetic nature and are controlled by a system of interacting genes, among which the *E* maturity loci play an important role. The best studied of these are *E1*, *E2*, *E3*, *E4*, and *E9* [8]. It has been shown that the combined contribution of allelic variation at the *E1*–*E4* loci can explain approximately 62–66% of the variation in flowering time [9]. In addition, an association has been established between allelic combinations at the *E1*–*E4* loci and soybean ecotypes, indicating the important role of these loci in shaping the crop’s geographic adaptation [9]. The *E1* locus is one of the central regulators of soybean photoperiodic flowering and is involved in suppressing the expression of the florigen genes *GmFT2a* and *GmFT5a* [10]. The *E2* locus encodes a GIGANTEA-like protein and is also involved in regulating the timing of flowering and maturity [11]. The *E3* and *E4* loci, which encode phytochrome A (PhyA) proteins (phyA3 and phyA2, respectively) are associated with the perception of light signals and particularly important in the controlling of photoperiod responses in soybean [12,13]. The *E7* locus also controls the timing of flowering and maturity, as well as photoperiod sensitivity; the dominant allele is associated with later flowering and maturation and greater sensitivity to long photoperiods compared to the recessive *e7* [14].

Thus, soybean adaptation to a given photoperiod is determined not by the action of a single locus, but by the combined effect of several components of the genetic system regulating flowering and maturity. In particular, the allelic variation at the *E1*, *E3*, *E4*, and *E7* loci plays a significant role in modulating photoperiod sensitivity and soybean adaptating to the long days conditions of high latitudes [6]. At the same time, the effect of individual alleles depends on their combination with other loci, the genetic background, and growing conditions. Accordingly, when breeding soybean for adaptation to high-latitude conditions, it is of particular interest not only to identify individual favorable alleles, but also to select their optimal multilocus combinations.

In traditional breeding, selection on flowering and maturation times is carried out mainly based on the phenotypic assessment of plants under field conditions [15,16]. However, phenotypical traits are significantly influenced by environmental conditions, and evaluating a large number of plants in segregating populations requires labor-intensive field observations. MAS makes it possible to select directly based on the allelic state of target loci already at the early stages of the breeding process, regardless of the phenotypic expression of the trait and environmental influence [17]. This approach is particularly promising in segregating F_2_ populations, in which recombination of parental alleles generates various allelic combinations, making it possible to identify individual plants carrying desirable combinations of target loci [15,18].

Various types of molecular markers, including SSR (Simple Sequence Repeat) and CAPS (Cleaved Amplified Polymorphic Sequence), have been developed to identify allelic combinations of genes associated with the photoperiod response and maturity timing in soybean [10,13,19,20,21,22,23]. Their use enables genotypic selection of individual plants in segregating populations at target loci. At the same time, the phenotypic expression of maturity gene alleles depends on their combination, genetic background, and environmental conditions. Therefore, the effectiveness of MAS must be confirmed by subsequent evaluation of the selected material under target growing conditions.

For the northern regions of Kazakhstan, the development of early-maturing soybean varieties capable of transitioning to flowering in a timely manner and completing maturation under conditions of long summer days and a limited growing season is of particular importance [24,25]. One of the major constraints to expanding soybean production in Northern Kazakhstan is the limited availability of cultivars combining early maturity, high productivity, and adaptation to the long-day conditions of high latitudes [24]. However, the possibilities of targeted selection of favorable multilocus combinations of genes involved in the photoperiod response in segregating soybean populations, followed by phenotypic assessment of their progeny under the conditions of Northern Kazakhstan, remain insufficiently studied. Integrating early molecular selection with field evaluation in the target environment may increase the efficiency of developing source material adapted to high latitude conditions.

In the present study, we hypothesized that MAS of individual F_2_ plants based on allele combinations at the *E1*, *E3*, *E4,* and *E7* loci, followed by field assessment of their progeny under the conditions of Northern Kazakhstan, would make it possible to identify breeding material combining a favourable allelic composition with early flowering and timely maturation.

Of particular interest was the use of introduced early-maturing parental forms as donors of the functionally significant recessive alleles *e1-nl*, *e3-fs*, and *e4-SORE-I*, with the aim of introgressing them into Kazakhstani breeding material and generating new combinations of alleles at the photoperiod response loci.

The study aimed to perform MAS of plants from three F_2_ soybean populations using SSR and CAPS markers for the *E1*, *E3*, *E4,* and *E7* loci, followed by evaluation of the progeny of the selected plants based on flowering and maturation times and the main breeding-relevant traits under the conditions of Northern Kazakhstan.

## 2. Materials and Methods

### 2.1. Plant Material and Development of F_2_ Populations

Three F_2_ soybean populations were used for this study. The populations were F10 (Zara × Zlata), F13 (Zara × Maple Presto), and U4 (Birlik KV × LMF). A total of 770 F_2_ plants were included in molecular genotyping and MAS: 210 plants from F10, 330 from F13, and 230 from U4. They were obtained by crossing parental forms carrying different allelic combinations of maturity and photoperiod response loci *E1*, *E3*, *E4*, and *E7*. The Kazakhstani soybean cultivars Zara and Birlik KV, characterized by white flower color, high productivity, and adaptability to local growing conditions, were used as maternal forms in F10 and F13, and U4, respectively (Table 1). The cultivars LMF, Maple Presto, and Zlata, which have purple flower color and carry different combinations of recessive alleles at the studied *E* loci, were selected as the paternal forms of F10, F13, and U4, respectively. According to preliminary field trials, the paternal forms were characterized by early maturity and fully completed their growing cycle under high latitude conditions (50–53 °N) [25], and also demonstrated resistance to pod cracking.

Hybridization of parental forms and the production of the F_1_ generation were carried out at the research and breeding site of the Oilseed Crops Laboratory at the Kazakh Research Institute of Agriculture and Plant Growing (KRIAPG), following a previously published protocol [15]. The three crosses were Zara × Zlata (F10), Zara × Maple Presto (F13), and Birlik KV × LMF (U4). The hybrid nature of the F_1_ plants was confirmed based on corolla color: plants with a purple coloration, conditioned by the dominant *W1* allele, were identified as F_1_ hybrid [26]. Seeds obtained by self-pollination of confirmed F_1_ plants were used to generate the corresponding F_2_ populations, which were subsequently used for molecular genotyping and MAS.

The origin of the parental cultivars, their main morphobiological characteristics, and the allelic states of the studied *E* loci are presented in Table 1.

### 2.2. Cultivation of F2 Populations and MAS

To perform MAS, three F_2_ populations were grown in 2024 at the research and breeding site of the Laboratory of Oilseed Crops of the KRIAPG, located in the foothill zone of Almaty region in southeastern Kazakhstan, at an elevation of 740 m above sea level [43°23′91.54″ N, 76°68′71.47″ E]. Each F_2_ population F10, F13, and U4 was sown separately in a four-row plot with an area of 25 m^2^.

In June 2024, at the third trifoliolate (V3) stage, plants with sufficient vegetative development for leaf sampling and subsequent propagation were selected for molecular analysis. Severely underdeveloped or weak plants were excluded at this stage. No selection was performed before genotyping based on flowering time, maturity, or other reproductive phenological traits. Each selected F_2_ plant received an identifier consisting of the population code (F10, F13, or U4) and its original individual plant number. These identifiers were retained throughout the study to maintain correspondence between molecular genotyping records and the derived F_3_ progenies. Young leaves of individual plants were collected for genomic DNA extraction.

A total of 770 individual F_2_ plants were selected for molecular genotyping: 210 plants from F10, 330 plants from F13, and 230 plants fromU4.

Following leaf sampling, the plants were maintained in the field until full maturity. Seeds from each F_2_ plant were harvested separately and stored under the corresponding identification number for subsequent progeny evaluation.

**Figure 1:**
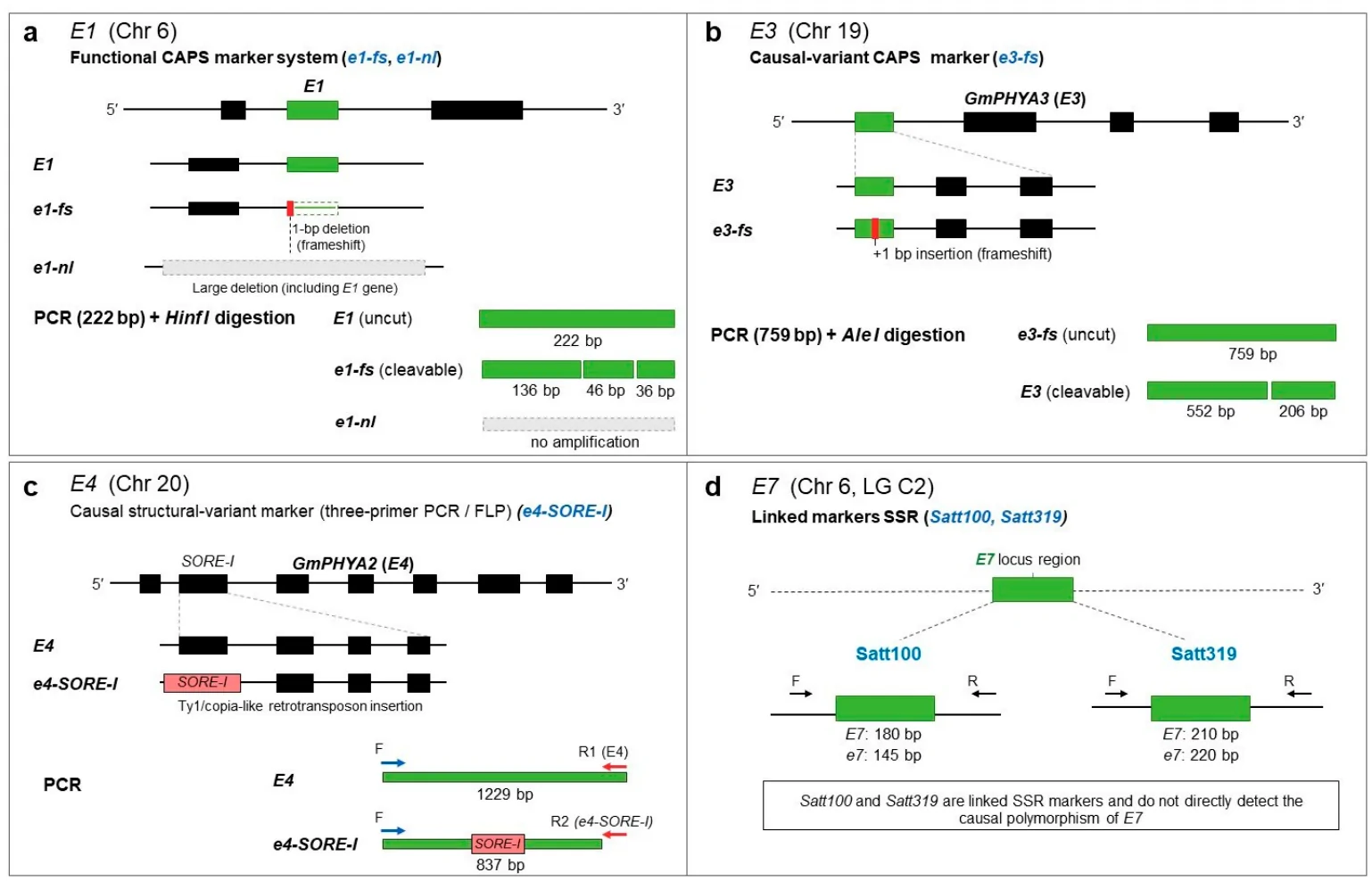
Schematic representation of molecular markers used for genotyping soybean ***E*** loci. (**a**) *E1* locus and the causal variants underlying *e1-fs* and *e1-nl*, with diagnostic PCR/CAPS profiles; (**b**) *E3* (*GmPHYA3*) locus and the *e3-fs* frameshift variant detected by CAPS analysis; (**c**) *E4* (*GmPHYA2*) locus and the *e4-SORE-I* structural variant detected using the three-primer PCR/FLP marker; (**d**) SSR markers Satt100 and Satt319 linked to the *E7* locus region and their associated marker allele sizes. Satt100 and Satt319 are linked markers and do not directly detect the causal polymorphism underlying *E7*. Expected diagnostic fragment sizes are indicated for each marker system.

Genomic DNA was extracted from young leaf tissue using the CTAB method [27]. Molecular genotyping was performed by PCR using an Eppendorf Mastercycler thermal cycler (Eppendorf, Hamburg, Germany). Functional PCR-based markers (CAPS, dCAPS, and FLP) for the *E1*, *E2*, *E3*, and *E4* loci, as well as linked SSR markers for the *E7* locus, were used for genotyping. All restriction endonucleases used for CAPS and dCAPS analyses were obtained from New England Biolabs (Ipswich, MA, USA). Gene IDs, targeted variants and their molecular consequences, marker types, marker–variant relationships, primer sequences, PCR conditions, restriction endonucleases, and diagnostic fragment sizes are provided in Supplementary Table S1. The molecular basis of the marker systems, the positions of the targeted causal variants or linked marker regions, and the corresponding diagnostic fragment patterns are schematically summarized in Figure 1.

PCR products and restriction digestion products were separated by electrophoresis on 8% polyacrylamide gel. Fragment sizes were estimated relative to the Step50 DNA molecular weight marker (Biolabmix LLC, Novosibirsk, Russia).

Based on the combined results of molecular genotyping, individual F_2_ plants were selected according to target alleles and their combinations. The selection criterion was defined separately for each hybrid population, taking into account the allelic composition of its parental forms. In F10, selection was based on the *e3-fs* allele of the *E3* locus; in F13, on the combination of target alleles at the *E3*, *E4*, and *E7* loci. In U4, selection was based on the *E1* and *E3* loci. The *E2* locus was not included as a differentiating criterion for MAS, since all parental forms carried the recessive *e2* allele. The *E1*/*e1-as* allelic state was likewise not used as a selection criterion in F10 and F13.

Seeds of individually selected plants were maintained separate for subsequent propagation and field evaluation.

### 2.3. Field Trials in the Northern Region of Kazakhstan

Field evaluation of the progenies of individual plants derived from the F_2_ populations F10, F13, and U4 was carried out in 2025 at the Zarechnoye experimental Station in Kostanay region, Northern Kazakhstan [53°22′60.50″ N, 63°78′66.24″ E].

The region has a continental climate, with cold winters and warm summers and a relatively short frost-free period of 110–130 days [28]. The mean annual air temperature is 4.2 °C, and mean annual precipitation is approximately 422 mm [29]. The soil at the experimental site is represented by southern medium loamy chernozems with a humus content of up to 3%. Weather conditions at the Zarechnoye experimental Station, (Kostanay Region) are presented in Table S2.

In 2025, 110 F_3_ progenies of individual F_2_ plants from three populations were formed for field evaluation: 30 progenies from F10, 56 from F13, and 24 from U4. Each F_3_ progeny retained the identification number of the original F_2_ plant, ensuring correspondence between its molecular genotyping results and the subsequent phenotypic evaluation of the progeny.

The field trial included both F_3_ progenies selected through MAS based on target alleles at the *E* loci, and additional progenies from the same hybrid populations selected by the breeder based on combination of agronomically valuable traits, including high productivity. Including this additional material enabled a comparative evaluation of progenies carrying different allelic combinations of the *E* loci under uniform conditions of Northern Kazakhstan.

Sowing was carried out on 20 May 2025. F_3_ progenies were grown without replication in single-row plots 1 m long. Twenty-five seeds of the corresponding progeny were sown per plot at a depth of 4 cm; inter-row spacing was 30 cm. The cultivar Ivushka (Kazakhstan) was used as the local standard check and was planted after every 10 test samples.

During the growing season, phenological observations of soybean growth and development were conducted according to a previously published methodology [30]. For each F_3_ progeny, we recorded two periods in days: from emergence to full flowering and from emergence to full maturity. Because the F3 progenies remained segregating, phenological stages were recorded at the progeny-plot level. Full flowering and full maturity were recorded when all plants within the corresponding progeny plot had reached the respective phenological stage. Considering the relatively short and variable frost-free period in the Kostanay Region (123–144 days based on long-term observations at the experimental site), 115 days from emergence to full maturity was used as an upper threshold indicating increased risk of exposure to early autumn frosts. Under Northern Kazakhstan conditions, approximately 8–10 days are required from sowing to emergence, and an additional 5–10-day period after full maturity is desirable to allow timely harvesting before the onset of unfavorable autumn weather. Accordingly, a more conservative threshold of 110 days from emergence to full maturity was applied for breeding selection to provide an adequate time margin for both crop establishment and harvesting within the frost-free period. Therefore, progenies completing maturation within 110 days were considered suitable for further breeding evaluation, provided that they also met the agronomic selection criteria. Progenies that did not complete maturation by the end of the growing season were recorded as immature and treated as a separate phenotypic category rather than as missing observations.

After reaching full maturity, yield component traits were evaluated according to the methodological guidelines [31].

### 2.4. Statistical Analysis

Statistical analyses were performed using JASP software, version 0.98.1 [32]. Phenological traits analyzed included the number of days from emergence to full flowering, the number of days from emergence to full maturity, and maturity status (matured or not matured). Days to maturity were analyzed only for progenies that reached full maturity, whereas maturity status was evaluated for all F_3_ progenies.

Descriptive statistics for quantitative traits included the number of observations (n), mean, standard deviation (SD), median, interquartile range (IQR), minimum, and maximum. Normality of the distributions within genotype classes was assessed using the Shapiro–Wilk test [33]. Because deviations from normality were detected in several genotype classes and group sizes were frequently small and unbalanced, non-parametric tests were used for comparisons of flowering and maturity times.

Differences among three or more independent genotype classes were evaluated using the Kruskal–Wallis test [34]. When the overall test was statistically significant, pairwise comparisons were performed using Dunn’s post hoc test [35], with Holm adjustment for multiple comparisons [36]. Rank epsilon-squared (ε^2^) was reported as the effect size for Kruskal–Wallis tests. Comparisons between two independent genotype classes were performed using the Mann–Whitney U test [37], with rank-biserial correlation (rrb) reported as the effect size.

Associations between categorical variables, including population or genotype class and maturity status, were evaluated using contingency-table analysis. Pearson’s χ^2^ test was applied where appropriate, with Cramer’s V reported as a measure of association strength [38]. For 2 × 2 contingency tables with small or zero cell counts, Fisher’s exact test was used.

Genotype–phenotype associations were evaluated at two complementary levels. First, all F_3_ progenies were characterized according to their multilocus *E1*–*E2*–*E3*–*E4*–*E7* profiles to describe variation in flowering time, maturity time, and the proportion of progenies reaching full maturity. Second, statistical comparisons were performed separately within each hybrid population to account for differences in genetic background. Accordingly, the association of *E3* with phenological traits was evaluated in F10; *E3*, *E4*, *E7*, and selected multilocus combinations were evaluated in F13; and *E1*–*E3* marker classes were compared in U4.

Multilocus genotype classes represented by very few progenies were summarized descriptively and were not subjected to unrestricted multiple pairwise testing. Targeted comparisons between biologically relevant multilocus classes were performed using the Mann–Whitney U test. The unresolved *e1-fs*/*e1-nl* class in U4 was retained as a marker-defined category and was not interpreted as a confirmed heterozygous genotype.

All statistical tests were two-sided, and statistical significance was accepted at *p* < 0.05. Exact *p*-values are reported where applicable; values below 0.001 are reported as *p* < 0.001.

## 3. Results

### 3.1. Molecular Identification and MAS of E1, E3, E4, and E7 Alleles in Soybean F2 Populations

#### 3.1.1. Identification of the *e1-fs* and *e1-nl* Alleles at the *E1* Locus

The *E1* locus was genotyped in the U4 population using the functional *e1-fs* CAPS marker and a presence–absence assay for the *e1-nl* deletion. The molecular basis and diagnostic patterns of these alleles are shown in Figure 1 and Supplementary Table S1 [10].

**Figure 2:**
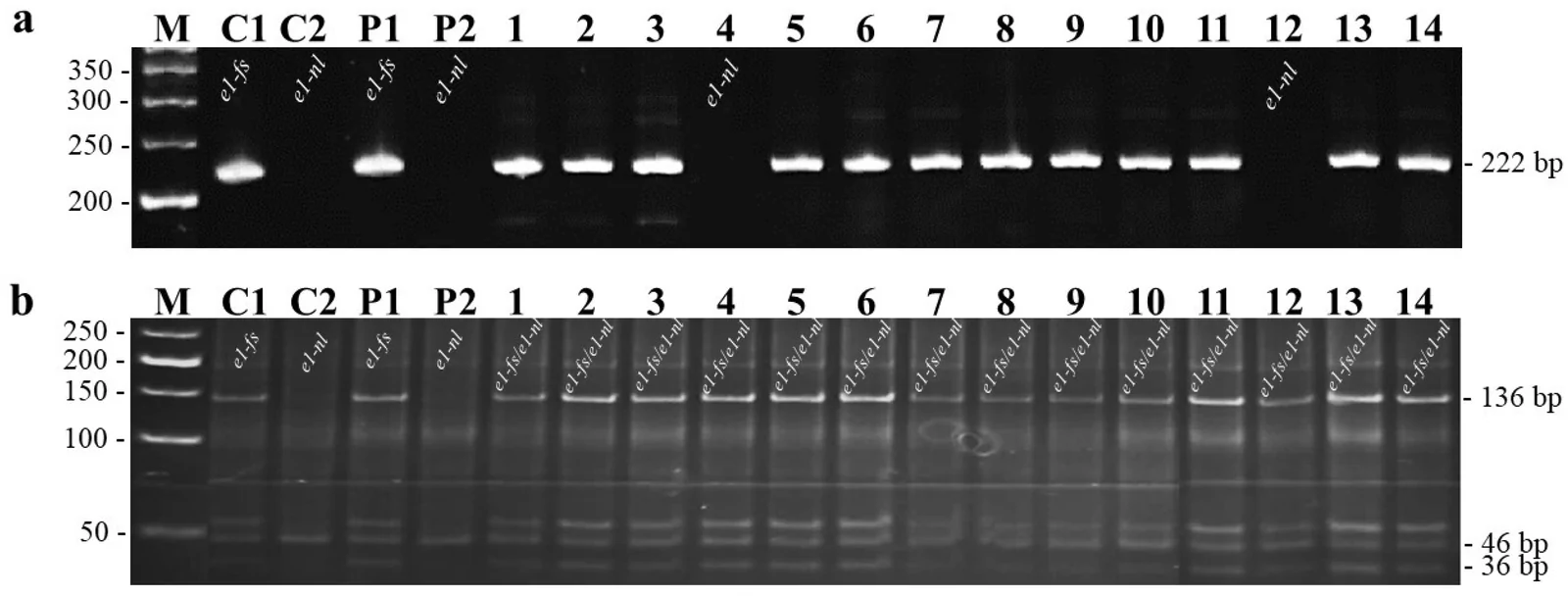
Identification of *E1* locus alleles in the F_2_ population U4 (Birlik KV × LMF) using the *e1-fs*/*e1-nl* marker assay. (**a**) PCR products before restriction; (**b**) PCR products after HinfI digestion M, Step50 DNA marker; C1, control genotype, Sakamotowase (*e1-fs*/*e1-fs*); C2, control genotype, Fiskeby V (*e1-nl*/*e1-nl*); P1, maternal cultivar Birlik KV; P2, paternal cultivar LMF; 1–14, representative individual F_2_ plants.

A total of 230 individual plants from the F_2_ population U4 (Birlik KV × LMF) were analyzed. No specific amplification was observed in 42 plants (18.3%), corresponding to the homozygous genotype *e1-nl*/*e1-nl* (Figure 2, Table 2). The remaining 188 plants (81.7%) produced an amplificon with the diagnostic *e1-fs* restriction pattern. Because the *e1-nl* allele does not produce an *E1*-specific amplicon, the assay could not distinguish *e1-fs*/*e1-fs* homozygotes from *e1-fs*/*e1-nl* plants. These 188 plants were therefore retained as an unresolved *e1-fs*/*e1-nl* marker class rather than assigned to a specific zygosity.

The parental forms of populations F10 and F13 also differed in their allelic states at the *E1* locus: the cultivar Zara carried the functional *E1* allele, whereas Zlata and Maple Presto carried the hypomorphic allele *e1-as*, characterized by partially reduced *E1* function [8]. However, the *E1*/*e1-as* allelic state was not used as a MAS criterion in these two populations. Therefore, targeted MAS at the *E1* locus was performed only in U4, whose parental cultivars Birlik KV and LMF carried *e1-fs* and *e1-nl*, respectively.

#### 3.1.2. Identification of the *e3-fs* Allele at the *E3* Locus

Genotyping to identify the *e3-fs* allele at the *E3* locus was performed in three F_2_ populations (F10, F13, and U4). The paternal cuitivars Zlata, Maple Presto, and LMF were the sources of the recessive *e3-fs* allele.

A total of 770 F_2_ plants were genotyped using the functional CAPS marker targeting the causal *e3-fs* variant in *GmPHYA3* [20]. The molecular basis of the allele and its diagnostic AleI restriction pattern are summarized in Figure 1 and Supplementary Table S1.

The homozygous *e3-fs*/*e3-fs* genotype was identified in 23 of 210 plants (10.9%) in F10, 42 of 330 (12.7%) in F13, and 26 of 230 (11.3%) in U4 (Table 2). The homozygous *E3/E3* genotype was detected in 43 (20.5%), 67 (20.3%), and 52 (22.6%) plants, respectively. The remaining 144 F10 (68.6%), 221 F13 (67.0%), and 152 U4 (66.1%) plants exhibited both digested and undigested fragments. Because this profile could represent either *E3*/*e3-fs* heterozygosity or incomplete restriction digestion, these plants were retained as an unresolved marker class and were not assigned a definitive genotype.

**Figure 3:**
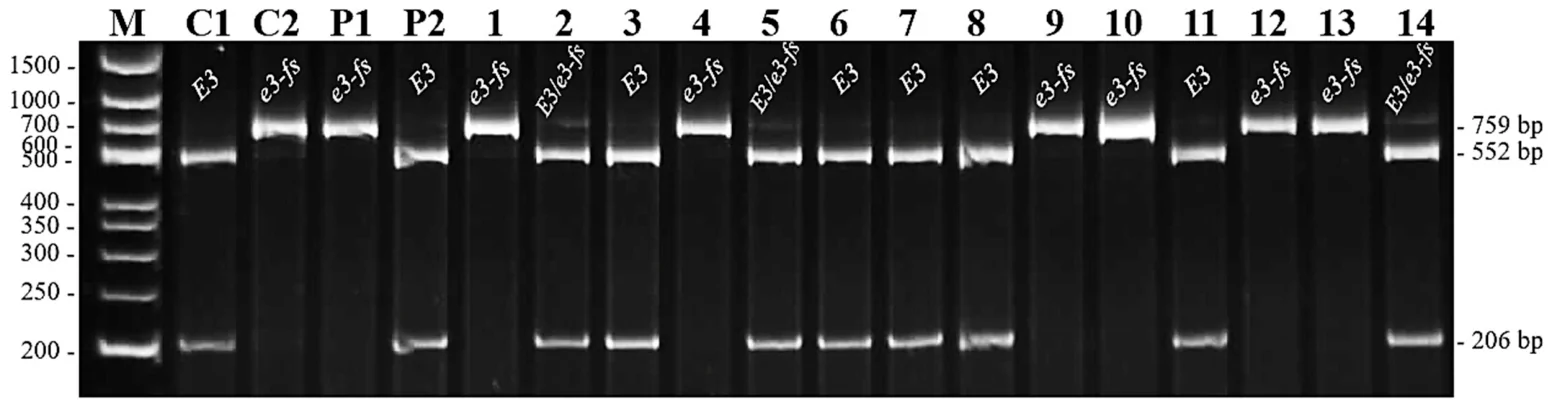
Representative CAPS profile for identification of the *e3-fs* allele at the *E3* locus in the F_2_ population F10 after AleI digestion. M, Step50 DNA marker; C1, control genotype, Harosoy (*E3*/*E3*); C2, control genotype, SibNIIK 315 (*e3-fs*/*e3-fs*); P1, maternal cultivar Zara; P2, paternal cultivar Zlata; 1–14, representative individual F_2_ plants.

Overall, 91 plants were unambiguously identified as *e3-fs*/*e3-fs* and retained for subsequent population-specific MAS: 23 from F10, 42 from F13, and 26 from U4 (Table 2). A representative CAPS profile is shown in Figure 3.

#### 3.1.3. Identification of the *e4-SORE-I* Allele at the *E4* Locus

The *E4* locus was genotyped in the F13 population using the functional allele-specific FLP marker targeting the causal *e4-SORE-I* insertion in *GmPHYA2* [21]. The molecular basis of the *e4-SORE-I* allele and its diagnostic amplification pattern are summarized in Figure 1 and Supplementary Table S1.

A total of 330 individual F_2_ plants were analyzed. The homozygous genotype *e4-SORE-I*/*e4-SORE-I* was detected in 78 plants (23.6% of all plants analyzed), the genotype *E4*/*E4* in 65 plants (19.7%), and the heterozygous genotype *E4*/*e4-SORE-I* in 159 plants (48.2%). In 28 (8.5%) plants, no specific amplification was observed, and they were excluded from further analysis of the *E4* locus.

**Figure 4:**
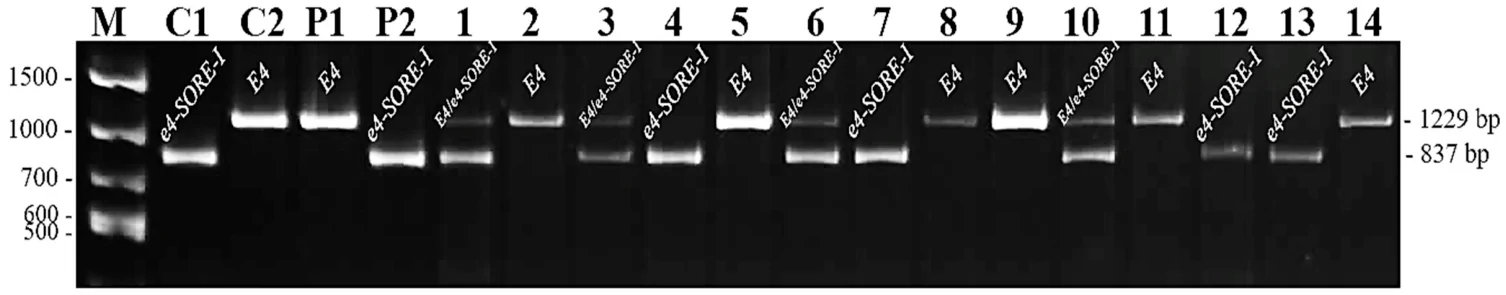
Representative FLP profile for identification of the *e4-SORE-I* allele at the *E4* locus in the F_2_ population F13. M, Step50 DNA marker; C1, control genotype, Fiskeby V (*e4-SORE-I*/*e4-SORE-I*); C2, control genotype, Harosoy (*E4E4*); P1, maternal cultivar Zara; P2, paternal cultivar Maple Presto; 1–14, representative individual F_2_ plants.

Among the 302 plants with an established genotype, the frequencies of *e4-SORE-I*/*e4-SORE-I*, *E4*/*e4-SORE-I*, and *E4*/*E4* were 25.8%, 52.6%, and 21.5%, respectively. This distribution was close to the expected 1:2:1 segregation ratio for a single locus in an F2 population. A representative FLP profile is shown in Figure 4.

#### 3.1.4. Identification of Allelic Variation Associated with the *E7* Locus

To analyze allelic variation associated with the *E7* locus in the F_2_ population F13, two SSR markers, Satt100 and Satt319, previously described as markers linked to the genomic region of the *E7* locus, were used [23]. These markers do not directly detect a causal *E7* variant; their genomic relationship to the *E7* locus and the marker alleles used in the present study are summarized in Figure 1 and Supplementary Table S1.

Control lines with known *E7* allelic states, OT94-47 (*e7*/*e7*) and Harosoy (*E7*/*E7*) were used to interpret the obtained electrophoretic profiles. In the parental cultivars, Satt100 produced 180- and 145-bp marker alleles in Zara (*E7*/*E7*) and Maple Presto (*e7*/*e7*), respectively. Classification based on Satt100 was independently verified using Satt319.

**Figure 5:**
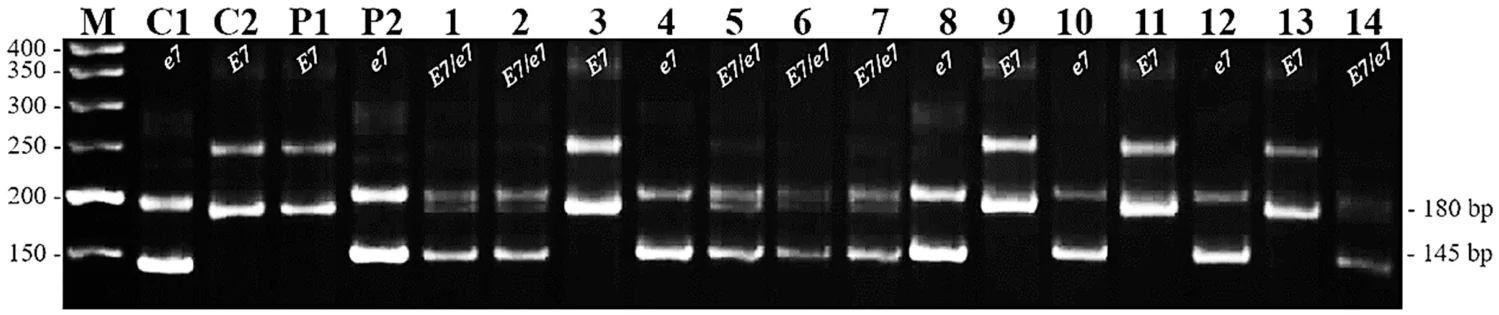
Representative Satt100 SSR profile associated with *E7* locus in the F_2_ population F13. M, Step50 DNA marker; C1, control genotype, OT94-47 (*e7*/*e7*); C2, control genotype, Harosoy (*E7*/*E7*); P1, maternal cultivar Zara; P2, paternal cultivar Maple Presto; 1–14, representative individual F_2_ plants.

A total of 330 individual F2 plants were analyzed. The Satt100 profiles associated with *E7*/*E7*, *E7*/*e7*, and *e7*/*e7* were detected in 78 (23.6%), 149 (45.2%), and 82 (24.8%) plants, respectively (Figure 5). No specific amplification was obtained in 21 plants (6.4%), which were excluded from further *E7*-associated marker analysis. Genotyping with Satt319 confirmed the Satt100-based classification, with no discrepancies between the two markers. Among the 309 plants with concordant marker genotypes, the corresponding frequencies were 25.2%, 48.2%, and 26.5%, respectively, and did not deviate from the expected 1:2:1 segregation ratio (χ^2^ = 0.50; df = 2; *p* = 0.78).

Thus, 82 F2 plants carrying marker profiles associated with the homozygous *e7*/*e7* state were retained for subsequent combined MAS (Table 2).

### 3.2. Combined MAS Based on the E-Locus Allelic Profiles

Based on the results of individual genotyping of F_2_ population plants, combined MAS was performed for target alleles at the photoperiod response loci. Selection criterion were defined separately for each hybrid population according to the allelic composition of its parental cultivars. The *E2* locus was not used as a differentiating criterion, since all parental forms carried the recessive *e2* allele.

In F10, targeted selection was performed at the *E3* locus, with Zlata serving as the source of the non-functional *e3-fs* allele. A total of 23 of 210 F_2_ plants (10.9%) were identified as *e3-fs*/*e3-fs* and selected for subsequent evaluation (Table 2). The other studied *E* loci were not used as differentiating MAS criteria in this population.

In F13, combined selection was performed at three loci *E3*, *E4*, and *E7*. Maple Presto was the source of the target recessive alleles *e3-fs*, *e4-SORE-I*, and *e7*, whereas Zara carried the corresponding dominant alleles *E3*, *E4*, and *E7*. Combined analysis of the individual genotyping data identified four F2 plants carrying *e3-fs*/*e3-fs* and *e4-SORE-I*/*e4-SORE-I* together with the SSR marker profile associated with *e7/e7*. An additional 26 plants combined *e4-SORE-I*/*e4-SORE-I* with the marker profile associated with *e7*/*e7* (Table 2). These plants were retained for subsequent propagation and field evaluation.

In U4, combined selection was performed at the *E1* and *E3* loci. Birlik KV carried *e1-fs* and *E3*, whereas LMF carried *e1-nl* and *e3-fs*. Combined analysis identified seven F2 plants carrying the homozygous *e1-nl*/*e1-nl* and *e3-fs*/*e3-fs* genotypes (Table 2). Given the parental allelic states at the other loci, these plants had the multilocus profile *e1-nl e2 e3-fs E4 e7*.

Thus, the applied MAS schemes enabled selection based on the genetic structure of each F_2_ population: a single target locus (*E3*) in F10, three loci (*E3*, *E4*, and *E7*) in F13, and two loci (*E1* and *E3*) in U4. The selected F_2_ plants were subsequently used to generate F_3_ progenies for field evaluation under Northern Kazakhstan conditions.

### 3.3. Field Evaluation of F_3_ Progenies Under the Conditions of Northern Kazakhstan

#### Phenological Evaluation of F_3_ Progenies

In 2025, a field evaluation of 110 F_3_ progenies derived from the three original hybrid populations, including 30 progenies from F10, 56 from F13, and 24 from U4 was carried out in the Kostanay Region. The studied material included both progenies of plants selected based on MAS results and additional breeding-valuable progenies from the same populations, representing various allelic combinations at the *E* loci.

The phenological response was evaluated based on the duration of the periods from emergence to full flowering and from emergence to full maturity. The local soybean cultivar Ivushka was used as the standart check. Under the experimental conditions, these periods were 35 and 105 days, respectively.

All 110 F_3_ progenies reached the full flowering. The period from emergence to full flowering ranged from 26 to 71 days. Mean flowering times were 53.5 days in F10, 47.2 days in F13, and 45.2 days in U4. The widest range was observed in F13 (26–67 days), whereas the corresponding ranges were 30–71 days in F10 and 31–69 days in U4 (Table S3). Overall, flowering time did not differ significantlly among the three original populations (Kruskal–Wallis, H(2) = 4.49; *p* = 0.106; ε^2^ = 0.041; Table S4).

Of the 110 F_3_ progenies evaluated, 80 (72.7%) reached full maturity, whereas 30 (27.3%) did not mature by the end of the growing season. The proportion of progenies reaching full maturity did not differ significantly among populations (χ^2^ = 5.94; df = 2; *p* = 0.051; Cramer’s V = 0.23). In F10, 19 of 30 progenies (63.3%) reached full maturity, compared with 39 of 56 (69.6%) in F13 and 22 of 24 (91.7%) in U4. The corresponding proportions of non-matured progenies were 36.7%, 30.4%, and 8.3%, respectively (Table S3).

Among the 80 progenies that reached full maturity, the period from emergence to maturity ranged from 90 to 125 days. The shortest mean period to maturity was recorded in U4 (111.4 days; range, 96–119 days), followed by F13 (114.7 days; range, 90–125 days) and F10 (117.9 days; range, 115–122 days) (Table S3). Days to maturity differed significantly among the three populations (Kruskal–Wallis, *H*(2) = 18.76; *p* < 0.001; rank ε^2^ = 0.237; Table S4). Dunn’s post hoc test with Holm correction showed significant differences between F10 and F13 (*p* = 0.021), F10 and U4 (*p* < 0.001), and F13 and U4 (*p* = 0.021).

**Figure 6:**
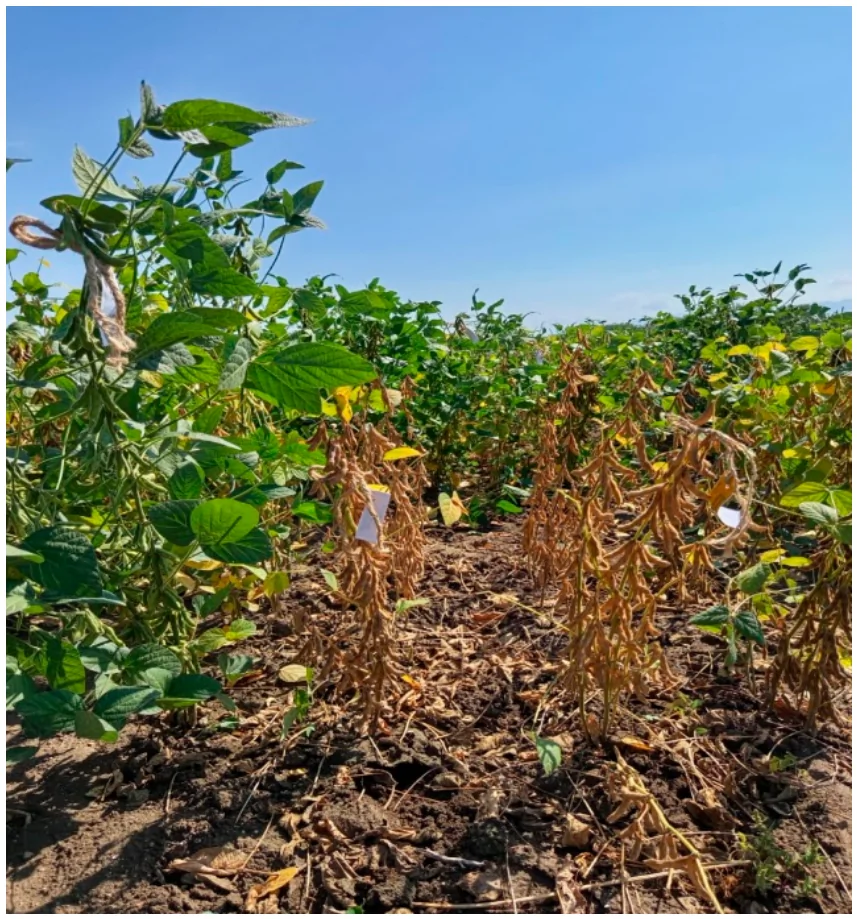
Contrasting maturity of soybean F_3_ progenies from F13 at harvest in Northern Kazakhstan (Kostanay Region): early-maturing progeny with the *E1*/*e1-as e2 e3-fs e4 e7* profile and late-maturing progeny with the *E1*/*e1-as e2 E3 E4*/*e4 e7* profile.

Based on the predefined maturity criteria, progenies requiring more than 115 days to reach full maturity were considered at increased risk of early autumn frost. Fourteen progenies (6 from F13 and 8 from U4) completed maturation within the conservative 110-day breeding threshold. They are selected for further breeding evaluation based on their phenological and agronomic performance (Table 3, Figure 6). The 14 selected progenies represented five multilocus *E*-profiles: *e1-fs*/*e1-nl e2 e3-fs E4 e7* (6), *E1*/*e1-as e2 e3-fs e4 e7* (3), *E1*/*e1-as e2 E3 e4 e7* (3), *e1-nl e2 E3 E4 e7* (1), and *e1-nl e2 e3-fs E4 e7* (1).

Among the 14 selected progenies, 8 exceeded the local standard cultivar Ivushka in the number of pods per plant, while 11 exhibited a determinate growth habit. Four progenies (F-13/70, U-4/112, U-4/120, and U-4/126) combined favorable *E*-locus allele combinations, early maturity, a determinate growth habit, and high productivity (Table 3).

### 3.4. Association of Marker Classes and Allelic Combinations at E Loci with Flowering and Maturity Time in F3 Progenies

#### 3.4.1. Multilocus *E1–E2–E3–E4–E7* Profiles

**Figure 7:**
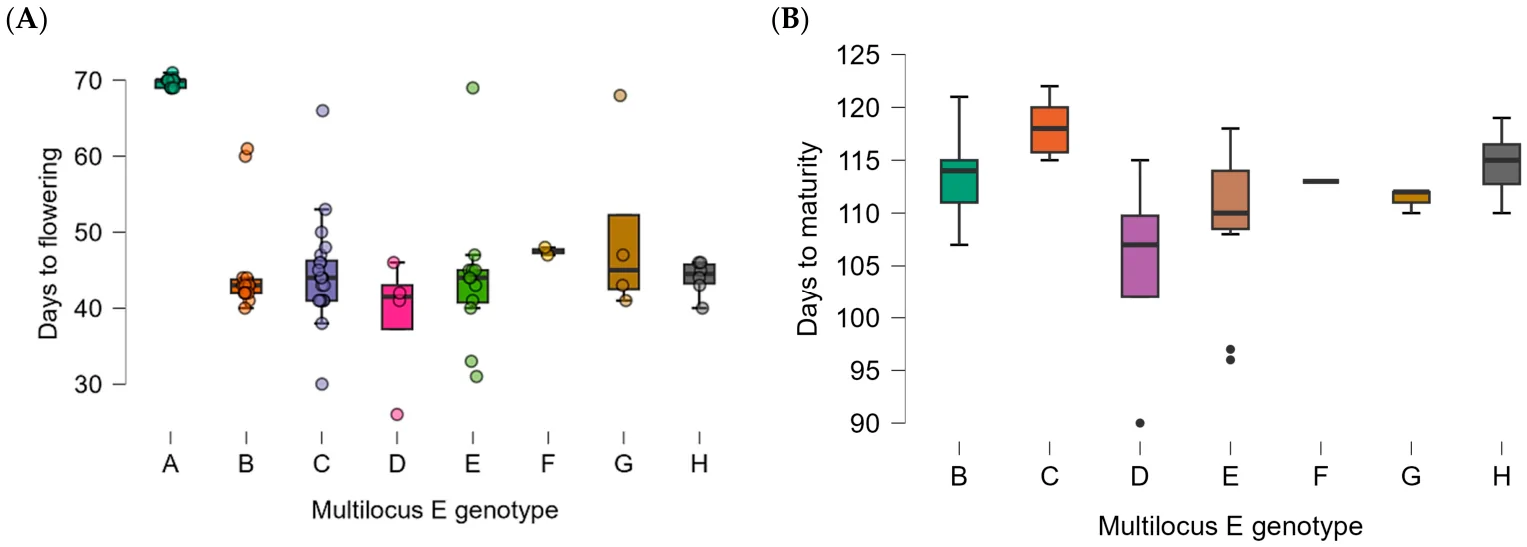
Flowering time and maturity characteristics of major multilocus *E*-locus profiles among F_3_ progenies under the conditions of Northern Kazakhstan (Kostanay Region). (**A**) Days from emergence to full flowering; (**B**) Days from emergence to full maturity among progenies that completed maturation: A, *E1*/*e1-as e2 E3 E4 E7;* B, *E1*/*e1-as e2 E3 e4-SORE-I e7;* C, *E1*/*e1-as e2 e3-fs E4 E7;* D, *E1*/*e1-as e2 e3-fs e4-SORE-I e7;* E, *e1-fs*/*e1-nl e2 e3-fs E4 e7;* F, *e1-nl e2 E3*/*e3-fs E4 e7;* G, *e1-nl e2 E3 E4 e7;* H, *e1-nl e2 e3-fs E4 e7.* In panel (**B**), maturity data are presented only for progenies that reached full maturity; all 11 progenies of profile A remained immature (Table 4). A relatively early phenological response was also observed for the profile *e1-fs*/*e1-nl e2 e3-fs E4 e7*. The mean flowering time was 43.9 days, and 11 of 12 progenies (91.7%) completed maturation, on average within 109.4 days. The profile *E1*/*e1-as e2 E3 e4 e7* also showed complete maturation (14/14 progenies), with a mean maturity period of 113.1 days. In contrast, several multilocus classes carrying dominant *E4* and/or *E7* alleles showed delayed or incomplete maturation (Table 4). Because several multilocus classes were represented by only a small number of progenies, these profile-level differences were interpreted descriptively.

Joint analysis of the multilocus *E1*–*E2*–*E3*–*E4*–*E7* profiles revealed substantial phenological differentiation among the progenies. The latest response was observed for the profile *E1*/*e1-as e2 E3 E4 E7*: all 11 progenies in this class flowered within a narrow range of 69–71 days (mean 69.7 days), and none of them reached full maturity (Table 4, Figure 7). In contrast, progenies with the profile *E1*/*e1-as e2 e3-fs e4 e7* showed the earliest mean flowering time (38.8 days), and all four progenies completed maturation, on average within 104.8 days.

#### 3.4.2. Analysis Within the F10 Population

**Figure 8:**
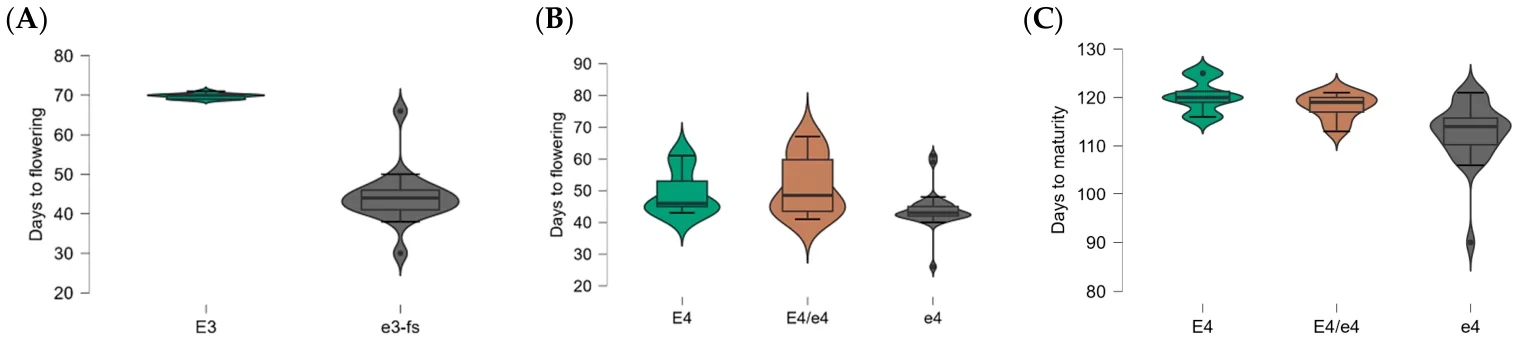
Association of *E*-locus genotypes with flowering and maturity time in F_3_ soybean progenies in Northern Kazakhstan (Kostanay Region). (**A**) Days to flowering according to *E3* genotype in F10; (**B**) Days to flowering according to *E4* genotype in F13; (**C**) Days to maturity according to *E4* genotype in F13. Days to maturity in panel (**C**) are shown only for progenies that reached full maturity.

In F10, the *E3* genotype was strongly associated with phenological response. Progenies with the *E3*/*E3* genotype flowered at a mean of 69.7 days (range, 69–71 days), whereas *e3-fs*/*e3-fs* progenies flowered at a mean of 44.2 days (range, 30–66 days; Figure 8A). The two genotype classes differed significantly in flowering time (Mann–Whitney U = 209.0; *p* < 0.001; |r| = 1.000; Table S3).

Differences in flowering time were accompanied by a contrasting ability to complete the growth cycle. All 19 *e3-fs*/*e3-fs* progenies reached full maturity, whereas none of the 11 *E3*/*E3* progenies completed maturation (Fisher’s exact test, *p* < 0.001; Cramer’s V = 1.000). Thus, within the F10 genetic background, the *E3* allelic state was associated with both flowering time and completion of maturation under the conditions of Northern Kazakhstan.

#### 3.4.3. Analysis Within the F13 Population

In F13, 39 of 56 progenies (69.6%) reached full maturity, whereas 17 (30.4%) did not reach maturity by the end of the growing season. Associations between phenological traits and marker classes differed among the analyzed loci. The allelic state of *E3* was not associated with flowering time (H(2) = 0.051; *p* = 0.975; ε^2^ < 0.001) or with maturity status (χ^2^(2) = 1.58; *p* = 0.453; Cramer’s V = 0.168). Among the 39 progenies that reached full maturity, days to maturity also did not differ among the *E3* genotype classes (H(2) = 1.86; *p* = 0.396; rank ε^2^ = 0.049; Table S4).

In contrast to *E3*, the *E4* locus was statistically significantly associated with flowering time, maturity status, and days to maturity. *e4-SORE-I*/*e4-SORE-I* progenies flowered at a mean of 43.9 days compared with 49.1 days for *E4*/*E4* and 51.7 days for *E4*/*e4-SORE-I* progenies (H(2) = 11.93; *p* = 0.003; ε^2^ = 0.217). After Holm correction, the homozygous recessive class differed significantly from both *E4*/*E4* (*p* = 0.048) and *E4*/*e4-SORE-I* (*p* = 0.005; Table S4).

Maturity status also differed significantly among the *E4* genotype classes (χ^2^(2) = 11.48; *p* = 0.003; Cramer’s V = 0.453). Full maturity was reached by 26 of 29 (89.7%) *e4-SORE-I*/*e4-SORE-I* progenies, compared with 9 of 18 (50.0%) *E4/e4-SORE-I* and 4 of 9 (44.4%) *E4/E4* progenies. Among the progenies that reached maturity, days to maturity differed significantly among *E4* genotype classes (*H*(2) = 11.41; *p* = 0.003; rank ε^2^ = 0.300; Table S4, Figure 8). Dunn’s post hoc test with Holm correction showed significant differences between *e4-SORE-I*/*e4-SORE-I* and both *E4/E4* (*p* = 0.027) and *E4/e4-SORE-I* (*p* = 0.027), whereas the latter two classes did not differ (*p* = 0.531).

For *E7*, differences in flowering time did not reach the predefined level of statistical significance (H(2) = 5.96; *p* = 0.051; ε^2^ = 0.108), although the mean time to flowering decreased from 51.5 days in *E7*/*E7* progenies to 45.7 days in *e7*/*e7* progenies.

In contrast, maturity status was significantly associated with the SSR marker classes linked to the *E7* locus (χ^2^(2) = 15.72; *p* < 0.001; Cramer’s V = 0.530). Full maturity was reached by 23 of 26 (88.5%) progenies with the marker profile associated with *e7/e7*, compared with 13 of 18 (72.2%) associated with *E7/e7* and 3 of 12 (25.0%) associated with *E7/E7*. Among the 39 progenies that reached maturity, days to maturity also differed among the *E7*-associated marker classes (*H*(2) = 10.19; *p* = 0.006; rank ε^2^ = 0.268; Table S4). After Holm correction, a significant pairwise difference was detected only between the *E7/e7*- and *e7/e7*-associated classes (*p* = 0.006); the other pairwise comparisons were not significant.

Among the multilocus classes of F13, the four progenies carrying the target combination *e3-fs/e3-fs e4-SORE-I/e4-SORE-I* with the SSR marker profile associated with *e7/e7* showed the earliest mean flowering and maturity times, 38.8 and 104.8 days, respectively. For the *E3/E3 e4-SORE-I/e4-SORE-I* class with the *e7/e7*-associated marker profile (*n* = 14), the corresponding values were 43.7 and 113.1 days, respectively. However, the differences between these two classes were not statistically significant for either flowering time (*U* = 37.0; *p* = 0.355) or maturity time (U = 44.0; *p* = 0.097). These results should be interpreted cautiously because of the small number of progenies carrying the triple recessive combination (Table S4).

#### 3.4.4. Analysis Within the U4 Population

In U4, no statistically significant differences in flowering time were found among the four *E1*–*E3* classes (H(3) = 3.87; *p* = 0.275; ε^2^ = 0.168). Mean values ranged from 43.9 days for the *e1-fs*/*e1-nl e3-fs* class to 49.8 days for the *e1-nl E3* class. Similarly, the E1–E3 classes did not differ significantly either in their ability to complete maturation (χ^2^(3) = 2.18; *p* = 0.536; Cramer’s V = 0.302) or in the duration of the period to full maturity among matured progenies (H(3) = 4.38; *p* = 0.223; ε^2^ = 0.209; Table S3).

Overall, the association of the *E* loci with phenological response depended on the original hybrid population. In F10, *E3* was strongly associated with flowering time and completion of maturation. The *e3-fs*/*e3-fs* progenies flowered earlier and maturated successfully. In F13, *E4* was significantly associated with flowering time, maturity status, and days to maturity. The *E7*-associated SSR marker classes were significantly associated with maturity status and days to maturity but not with flowering time. No significant associations were detected between the *E1–E3* classes and the evaluated phenological traits in U4. Among the multilocus classes in F13, the target combination *e3-fs/e3-fs e4-SORE-I/e4-SORE-I*, with the SSR marker profile associated with *e7/e7* was characterized by the earliest mean flowering and maturity times.

## 4. Discussion

Soybean adaptation to high-latitude conditions is determined by the combination of alleles at the photoperiod response loci and their phenotypic expression under specific growing conditions. In this study, MAS in F_2_ populations was combined with subsequent field evaluation of F_3_ progenies in Northern Kazakhstan. The results obtained revealed substantial differences in the phenotypic expression of the *E* loci among populations. This shows value of considering multilocus *E*-locus profiles when selecting early-maturing, photoperiod-adapted breeding material for high-latitude conditions.

### 4.1. Effectiveness of MAS for Developing Photoperiod-Adapted Material

The effectiveness of MAS largely depends on the selection of parental forms contrasting in the target alleles. This is part of a modern strategy for high-latitude breeding [39]. In the present study, the early-maturing cultivars LMF, Maple Presto, and Zlata were used as donors of the valuable non-functional alleles *e1-nl*, *e3-fs*, and *e4-SORE-I* for their introduction into Kazakhstani breeding material. All parental forms carried the recessive *e2* allele, so the *E2* locus was not used as a differentiating MAS criterion. A high frequency of *e2* is typical of early-maturing soybean germplasm. Yao et al. (2024) [39] identified *e2-ns* in 92% of the Chinese and European elite cultivars studied and demonstrated its association with earlier flowering and maturation. Zara and the paternal forms Maple Presto and Zlata differed at the *E1* locus (*E1* and *e1-as*). *E1* was not used as an MAS criterion in populations F10 and F13. This is consistent with the findings of Yao et al. (2024), according to which *e1-as* did not affect flowering time compared with functional *E1* [39].

Molecular markers have been developed for the main maturity and photoperiod response loci *E1*, *E2*, *E3*, *E4*, and *E7*. They allow targeted selection for these loci in segregating populations [10,19,20,21,23]. However, flowering and maturation times are determined not only by individual loci but also by their allelic combinations and genetic background [40]. In this work, individual genotyping of F_2_ plants allowed selection based on the allelic composition of each hybrid population and the identification of plants carrying the target multilocus combinations. This is consistent with Liu et al. (2020) [41], who showed an association between *E1*–*E4* combinations and flowering time, maturation time, and geographic adaptation in soybean.

The effectiveness of combining MAS with subsequent field evaluation has previously been demonstrated for Kazakhstani soybean breeding material. Selection at the *E1*, *E3*, *E4*, and *E7* loci followed by testing at 53° N identified early-maturing lines with various *E*-allele combinations. These lines completed their growth cycle within 92–102 days [15]. Here, the approach was extended to individual F_2_ plant selection. We used functional markers for molecularly characterized *E*-locus variants, together with linked SSR markers for *E7*, and then evaluated their F_3_ progenies. The target marker classes were expressed differently among populations. This highlight the importance of the multilocus genetic context for photoperiod adaptation [42].

Thus, the applied marker systems enabled targeted selection of F_2_ plants carrying the desired ***E***-locus alleles and marker profiles. They also allowed the development of F_3_ progenies with new multilocus combinations. At the same time, the results highlight the importance of combining MAS with field evaluation in the target growing region to identify progenies in which favorable marker profiles are accompanied by early flowering and timely completion of maturation.

### 4.2. Dependence of the Phenotypic Effect of the E3 Locus on Genetic Background

A strong association between *E3* allelic state and phenological response was observed in F10. The *e3-fs*/*e3-fs* progenies flowered substantially earlier than *E3/E3* progenies and completed maturation. The *E3/E3* progenies did not reach full maturity. This agrees with the known function of *E3*, which corresponds to the *GmPHYA3* gene. Functional *E3* increases soybean sensitivity to LD and delays the transition to flowering. Non-functional alleles, including *e3-fs*, are associated with reduced photoperiod sensitivity and earlier development [20,43].

However, the phenotypic association with *E3* differed among populations. In F13, *E3* showed no independent association with flowering time, maturity status, or days to maturity among progenies that reached full maturity. In this population, *E4* and *E7*-associated marker classes also segregated, and *E4* was significantly associated with flowering time, maturity status, and days to maturity. In U4, *e3-fs* co-segregated with different marker classes at *E1*. No statistically significant differences in flowering time, maturity status, or days to maturity were found among the *E1*–*E3* classes. Because the *E1* and *E3* states varied jointly, the independent effect of *e3-fs* could not be assessed.

The F13 result agrees with findings that the phenotypic expression of *E3* depends on its combination with other maturity loci. Different combinations of *E1*, *E3*, and *E4* produce different levels of photoperiod sensitivity, both before and after flowering [20]. Broad genotyping of soybean cultivars has also demonstrated that flowering time, maturation time, and latitudinal adaptation are determined by combinations of *E1*–*E4* alleles rather than by a single locus [40,41].

Thus, across the three populations, *e3-fs* should not be regarded as a universal marker of early maturity with a uniform effect in all breeding combinations. Its association with phenological response depends on the genetic context in which the allele occurs, so *E3* should be evaluated as part of multilocus combinations.

### 4.3. Associations of E4 and E7 with Flowering and Maturation

In F13, *E4* was consistently associated with flowering time, completion of maturation, and days to maturity among progenies that reached full maturity. The homozygous *e4-SORE-I* state was associated with earlier flowering, a higher proportion of progenies reaching full maturity, and earlier maturation.

This agrees with the known function of *E4*, which corresponds to the PHYA gene *GmPHYA2*. The *e4-SORE-I* allele contains a retrotransposon insertion that disrupts *E4* function and reduces sensitivity to LD [22]. Non-functional *E4* alleles are considered an important component of soybean adaptation to high-latitude LD conditions, especially in combination with recessive variants at other *E* loci [20,22].

The SSR marker classes associated with *E7* showed a different pattern. Differences in flowering time were not significant. However, these classes were significantly associated with both completion of maturation and days to maturity among matured progenies. *E7* was originally identified as a locus controlling flowering, maturation, and photoperiod sensitivity. In isogenic lines, *E7*/*E7* delayed flowering and maturation compared with *e7*/*e7*. The magnitude of the effect depended on day length and light spectral composition [14,44]. Under natural and extended photoperiods, the *e7*/*e7* line matured approximately six days earlier than the corresponding *E7*/*E7* line [44].

The differing expression patterns for *E4* and *E7*-associated marker classes suggest that the influence of photoperiod response loci extend beyond the timing of the transition to flowering. Photoperiod may also regulate subsequent stages of reproductive development. The combination of alleles at several *E* loci determines the overall duration of the growing season [20,44]. Therefore, the association of *e4-SORE-I* with flowering and maturation traits, together with the association of *E7*-linked SSR marker classes predominantly with maturation-related traits, may reflect different contributions of these genomic regions to adaptive phenology under the LD conditions of Northern Kazakhstan. The again highlight the importance of multilocus *E*-locus profiles for evaluating soybean adaptation to high latitudes.

### 4.4. Multilocus E Combinations and Early Maturity

In F13, progenies carrying the target combination *e3-fs*/*e3-fs e4-SORE-I*/*e4-SORE-I e7*/*e7*, with the SSR marker profile associated with *e7/e7*, had the earliest mean flowering and maturation times. Because this class had a small number of progenies, differences from other multilocus classes could not to be statistically confirmed. The observed pattern is consistent with data on the accumulation of non-functional *E*-locus alleles in early-maturing cultivars adapted to high latitudes [39,40,41]. Liu et al. (2020) [41] showed that cultivars carrying mutations at all four studied loci (*E1–E4*) were flowered and matured very early. They were predominantly distributed in cold, high-latitude regions.

Our data also suggest that early maturity is not necessarily associated with the maximum number of recessive *E*-locus alleles. In U4, early maturation was observed in progenies carrying *e1*, *e3-fs*, and the *e7*-associated marker profile together with functional *E4*. A similar pattern was previously observed in Northern Kazakhstan, where the *e1e3E4e7* combination was characterized by the earliest flowering and maturation among the genotypes studied [15]. This is consistent with Jiang et al. (2014) [40], who showed that different combinations of functional and non-functional *E*-locus alleles are associated with adaptation to different latitudinal conditions.

Considerable phenotypic variability may persist even within a single multilocus class. Such variability was noted both in the present study, among the F_3_ progenies, and previously among cultivars carrying identical *E1*–*E4* combinations [41,45,46]. This suggests that additional loci and the genetic background also contribute to the regulation of flowering and maturation. Consequently, multilocus selection based on known *E* genes may help narrow the search for potentially adaptive genotypes. However, identification of early-maturing breeding material still requires phenotypic evaluation in the target growing environment.

### 4.5. Breeding Significance of the Results for Northern Kazakhstan

MAS followed by field evaluation identified 14 F_3_ progenies from F13 and U4 that completed maturation within 90–110 days and met the additional agronomic selection criteria. These progenies carried target *E*-locus alleles or marker profiles identified through molecular genotyping.

In contrast, the additional progenies selected by the breeder mainly for high productivity in the Almaty Region did not meet the ≤110-day maturity criterion in Northern Kazakhstan. They were not retained for further breeding. Although the selected progenies did not uniformly surpass the local cultivar Ivushka in earliness. Howerver, they completed maturation within the defined breeding threshold and represented diverse *E*-locus profiles combined with agronomically valuable traits. This result highlights the value of combining MAS with field evaluation in the target environment, rather than relying on productivity-based selection alone. Notably, four selected F_3_ progenies combined favorable *E*-locus profiles with a determinate growth habit and high productivity.

### 4.6. Study Limitations and Future Directions

In contrast to the SSR markers used previously [15], the use of functional PCR-based markers in the present study provided direct identification of molecularly characterized allelic variants at the *E1*, *E3*, and *E4* loci, including *e1-nl*, *e1-fs*, *e3-fs*, and *e4-SORE-I*. This facilitated targeted selection for these alleles in the segregating breeding material.

CAPS analysis, however, has technical limitations related to the additional restriction-digestion and electrophoretic separation steps. In some cases, incomplete restriction digestion complicated the interpretation of heterozygous profiles. For the *e1-nl/e1-fs* combination, heterozygous plants could not to be unambiguously distinguished from plants homozygous for *e1-fs*. A promising direction is the transition to high-throughput KASP genotyping. Allele-specific KASP markers have already been developed and validated for several functionally important variants at *E1*–*E4* [41].

Excluding severely underdeveloped plants before genotyping may have introduced some selection bias into the analyzed F_2_ populations.

Another limitation concerns the structure of the field experiment. The F_3_ progenies represented still-segregating breeding material. Each progeny’s marker profile was assigned from its corresponding F_2_ parental plant. Consequently, progenies from heterozygous F_2_ plants could keep segregating at the respective *E* loci. The observed genotype–phenotype associations should not be interpreted as effects of genetically fixed lines. In addition, some multilocus classes were represented by small numbers of progenies, limiting the statistical power for assessing inter-locus interactions. The field trial was conducted at a single site in Northern Kazakhstan, without replication, owing to the limited number of seeds obtained from individual F_2_ plants. Therefore, the associations identified between marker classes and phenological traits require confirmation in subsequent fixed generations, in multi-year trials, and, where possible, in several environments. Further work will focus on the fixation of promising allelic combinations, on higher-throughput genotyping, and on evaluation of the stability of early maturity and productivity in the selected material.

## 5. Conclusions

MAS enabled identification of target alleles at the *E1*, *E3*, and *E4* loci and marker profiles associated with the *E7* locus. It also allowed development of new multilocus profiles in three soybean hybrid populations.

The phenotypic associations of the studied loci differed among the genetic backgrounds. In F10, *e3-fs* was strongly associated with earlier flowering and completion of maturation. In F13, *e4-SORE-I* was associated with earlier flowering, a higher frequency of completed maturation, and earlier maturity. *E7*-associated SSR marker classes were significantly associated with maturity status and days to maturity. The target combination *e3-fs/e3-fs e4-SORE-I/e4-SORE-I*, with the SSR marker profile associated with *e7/e7*, was characterized by the earliest mean flowering and maturation times among the multilocus classes in F13.

Based on the field evaluation, we selected 14 F_3_ progenies from F13 and U4 for further breeding work. They are reached maturity within 90–110 days and met additional agronomic selection criteria were selected. Four F_3_ progenies combined favorable *E*-locus profiles with a determinate growth habit and high productivity.

Hybridization and MAS enabled targeted selection of breeding material carrying the valuable alleles *e1-nl*, *e3-fs*, and *e4-SORE-I* and favorable multilocus profiles. The results obtained highlight the value of combining multilocus MAS with field evaluation in the target environment for developing early-maturing, photoperiod-adapted soybean breeding material for Northern Kazakhstan.

## Figures and Tables

**Table 1:** Origin, morphobiological characteristics, and allelic states of maturity loci in the soybean parental forms used to develop the F_2_ populations (mean data for 2018–2025).

Name of Variety	Origin	M or F	Color of Flower	Vegetation Period (Days)	Maturity Group	*E1*	*E2*	*E3*	*E4*	*E7*	Growth Type	Seed Yield in Plot (g m^−2^)	TSW (g)
Birlik KV	Kazakhstan	♀	white	116	I	*e1-fs*	*e2*	*E3*	*E4*	*e7*	indetermin.	391.6	174.2
Zara	Kazakhstan	♀	white	117	I	*E1*	*e2*	*E3*	*E4*	*E7*	determin.	474.4	172.8
LMF	Poland	♂	purple	87	000	*e1-* *nl*	*e2*	*e3-fs*	*E4*	*e7*	determin.	338.6	174.8
Maple Presto	Canada	♂	purple	90	00	e1-as	*e2*	*e3-fs*	*e4-SORE-I*	*e7*	indetermin.	273.4	180.1
Zlata	Russia	♂	purple	93	00	e1-as	*e2*	*e3-fs*	*E4*	*E7*	determin.	194.2	172.1

**Table 2:** Summary of MAS in three soybean F_2_ populations based on *E1*, *E3*, *E4*, and *E7* loci.

Populations	The Total Number of Plants	Individual Loci				Combination of Loci		
		*e1-nl/* *e1-nl*	*e3-fs*	*e4-SORE-I*	*e7*	*e3-fs e4-SORE-I e7*	*e4-SORE-I e7*	*e1-nl e3-fs*
F10 (Zara × Zlata)	210	-	23	-	-			
F13 (Zara × Maple Presto)	330	-	42	78	82	4	26	-
U4 (Birlik KV × LMF)	230	42	26	-	-		-	7
Total	770	42	91	78	82	7	26	7

**Table 3:** The F_3_ progenies selected for further breeding based on field evaluation in Northern Kazakhstan.

Population	Line ID	Multilocus *E* Profile	Days to Flowering	Days to Maturity	The Number of Beans per Plant, pcs	Growth Type
Ivushka	St.	*e1-as e2 e3-fs E4 E7*	35	105	88.5 ± 25.3	indetermin.
F13	F-13/70	*E1*/*e1-as e2 e3-fs e4 e7*	26	90	94.7 ± 15.2	determin.
	F-13/72	*E1*/*e1-as e2 e3-fs e4 e7*	41	106	87.8 ± 12.8	determin.
	F-13/164	*E1*/*e1-as e2 e3-fs e4 e7*	42	108	83.4 ± 21.4	determin.
	F-13/177	*E1*/*e1-ase2E3e4e7*	43	110	75.2 ± 16.3	determin
	F-13/204	*E1*/*e1-ase2E3e4e7*	43	107	101.2 ± 12.7	indetermin.
	F-13/205	*E1*/*e1-ase2E3e4e7*	42	107	96.2 ± 25.2	determin.
U4	U-4/4	*e1-nl e2 E3 E4 e7*	41	110	48.5 ± 20.1	determin.
	U-4/16	*e1-nl e2 e3-fs E4 e7*	45	110	87.5 ± 15.3	determin.
	U-4/112	*e1-fs*/*e1-nle2e3-fsE4e7*	31	97	115.4 ± 10.2	determin.
	U-4/120	*e1-fs*/*e1-nle2e3-fsE4e7*	41	96	124.5 ± 15.8	determin.
	U-4/125	*e1-fs*/*e1-nle2e3-fsE4e7*	40	110	130.8 ± 20.6	indetermin
	U-4/126	*e1-fs*/*e1-nle2e3-fsE4e7*	33	108	120.2 ± 12.8	determin.
	U-4/136	*e1-fs*/*e1-nle2e3-fsE4e7*	45	109	84.0 ± 5.9	determin.
	U-4/144	*e1-fs*/*e1-nle2e3-fsE4e7*	44	110	173.7 ± 21.7	indetermin.

**Table 4:** Phenological characteristics and maturity status of the major multilocus classes of F_3_ progenies under Northern Kazakhstan conditions.

Multilocus Profile *E1*–*E2*–*E3*–*E4*–*E7*	Total, *n*	Flowering, Mean ± SD, Days	Median	Matured, n (%)	Maturation, Mean ± SD, Days ^1^	Median, Days	Min–Max, Days
Ивушка (стандарт)	-	35	-	-	105	-	
*E1*/*e1-as e2 E3 E4 E7*	11	69.7 ± 0.6	70	0 (0)	-	-	-
*E1*/*e1-as e2 E3 e4 e7*	14	43.7 ± 5.1	42.5	14 (100)	113.1 ± 3.8	114	107–121
*E1*/*e1-as e2 e3-fs E4 E7*	20	44.6 ± 6.9	44	20 (100)	117.9 ± 2.4	118	115–122
*E1*/*e1-as e2 e3-fs e4 e7*	4	38.8 ± 8.8	41.5	4 (100)	104.8 ± 10.6	107	90–115
*e1-fs*/*e1-nl e2 e3-fs E4 e7* ^2^	12	43.9 ± 9.3	44	11 (91.7)	109.4 ± 7.0	110	96–118
*e1-nl e2 E3*/*e3-fs E4 e7*	2	47.5 ± 0.7	47.5	2 (100)	113.0 ± 0.0	113	113–113
*e1-nl e2 E3 E4 e7*	4	49.8 ± 12.4	45	3 (75.0)	111.3 ± 1.2	112	110–112
*e1-nl e2 e3-fs E4 e7*	6	44.0 ± 2.3	44.5	6 (100)	114.7 ± 3.3	115	110–119

Note: ^1^ Maturity time values were calculated only for progenies that reached full maturity. ^2^ *e1-fs*/*e1-nl* denotes an unresolved marker class comprising the possible genotypes *e1-fs*/*e1-fs* and *e1-fs*/*e1-nl*, which could not be distinguished by the CAPS analysis used; this designation should not be interpreted as a confirmed heterozygous genotype.

## Data Availability

The original contributions presented in this study are included in this article and its Supplementary Materials. Further inquiries can be directed to the corresponding author.

## Supplementary Materials

The following supporting information can be downloaded at: https://www.mdpi.com/article/10.3390/life16101663/s1, Supplementary Material S1: Table S1. Molecular markers, target variants, primer sequences, PCR conditions, and diagnostic fragment sizes used for genotyping soybean maturity loci; Supplementary Material S2: Table S2. Air temperature and precipitation during the 2024 soybean field experiment at KRIAPG (Almaty Region) and the 2025 field experiment at the Zarechnoye Agricultural Experimental Station (Kostanay Region), Kazakhstan; Table S3. Descriptive statistics for days to flowering and maturity of soybean F3 progenies from the F10, F13, and U4 populations under field conditions in the Kostanay Region, Northern Kazakhstan; Table S4: Statistical analysis of associations between E-locus marker classes and flowering- and maturity-related traits in F3 soybean progenies.

## Abbreviations

The following abbreviations are used in this manuscript:

CAPS	Cleaved Amplified Polymorphic Sequence
CTAB	cetyltrimethylammonium bromide
dCAPS	derived Cleaved Amplified Polymorphic Sequence
FLP	Fragment Length Polymorphism
KASP	Kompetitive Allele-Specific PCR
KRIAPG	Kazakh Research Institute of Agriculture and Plant Growing
LD	Long-day
MAS	Marker-Assisted Selection
PCR	Polymerase Chain Reaction
PhyA	Phytochrome A
SSR	Simple Sequence Repeat
