# Supplementary material for: Marker-Assisted Selection of Soybean E-Locus Alleles Using Functional and Linked Markers: Phenotypic Validation Under High-Latitude Conditions
Source: Life (Basel). 2026 Oct 3;16(10):1663. doi: 10.3390/life16101663 (PMC13705672; doi:10.3390/life16101663)
Supplement: Supplementary file 1 [file life-16-01663-s001.zip › Supplementary S2-Tables S2-S4.pdf]

life MDPI

Supplementary Materials S2

Marker-Assisted Selection of Soybean *E*-Locus Alleles Using Functional and Linked Markers: Phenotypic Validation under High-Latitude Conditions

Maikotov, B.; Zhumaliyeva, G; Didorenko, S.; Kushanova, R.; Zinchenko, A.; Bastaubayeva, S.; Ahmed, Z.; Yerzhebayeva, R.

Table S2: Air temperature and precipitation during the 2024 soybean field experiment at KRIAPG (Almaty Region) and the 2025 field experiment at the Zarechnoye Agricultural Experimental Station (Kostanay Region), Kazakhstan.

		Air temperature, °C		Precipitation, mm	
Month	Year	Mean monthly temperature	Multi-year average	Monthly	Multi-year average
KRIAPG in Almaty Region					
April	2024	12.8	10.9	111.3	110.6
May		17.6	16.3	121.2	98.4
June		24.5	21.2	19.7	59.9
July		25.0	23.7	85.2	56.9
August					
AES "Zarechnoye" in the Kostanay Region					
April	2025	11,4	5,3	38,8	26,0
May		16,2	13,7	36,9	36,0

June	20,6	20,0	33,6	35,0
July	20,4	20,9	53,7	56,0
August	18,9	18,9	48,5	35,0
September	13,4	12,5	50,4	25,0

https://doi.org/10.3390/life16101663

Table S3. Descriptive statistics for days to flowering and maturity of soybean F3 progenies from the F10, F13, and U4 populations under field conditions in the Kostanay Region, Northern Kazakhstan.

Statistic	Days to flowering			Days to maturity		
	F10	F13	U4	F10	F13	U4
Valid	30	56	24	19	39	22
Missing	0	0	0	11	17	2
Mode	43.53	43.20	44.78	115.5	115.6	112.1
Median	46.50	45.00	44.50	119.0	115.0	112.0
Mean	53.53	47.23	45.21	117.9	114.7	111.4
Std. deviation	13.63	7.954	8.246	2.505	6.117	5.620
IQR	26.00	5.750	3.750	4.500	8.00	5.000
Shapiro–Wilk	0.806	0.836	0.757	0.873	0.871	0.822
p-value of Shapiro–Wilk	<.001	<.001	<.001	0.016	<.001	0.001
Minimum	30.00	26.00	31.00	115.0	90.00	96.00
Maximum	71.00	67.00	69.00	122.0	125.0	119.0

Note: For days to maturity, missing values correspond to progenies that did not reach full maturity by the end of the growing season (F10, n = 11; F13, n = 17; U4, n = 2). Descriptive statistics for days to maturity were calculated only for progenies that reached full maturity.

Table S4. Statistical analysis of associations between *E*-locus marker classes and flowering- and maturity-related traits in F3 soybean progenies.

A. Overall tests

**Population**	**Trait**	**Factor**	**Test**	**Statistic**	**df**	***p***	**Effect size**
All	Maturity status	Population	Pearson χ^2	5.944	2	0.051	Cramer's V = 0.232
All	Days to flowering	Population	Kruskal–Wallis	H = 4.493	2	0.106	Rank ε^2 = 0.041
All	Days to maturity	Population	Kruskal–Wallis	H = 18.76	2	<0.001	Rank ε^2 = 0.237
F10	Maturity status	*E3*	Fisher's exact	—	—	<0.001	Cramer's V = 1.000
F10	Days to flowering	*E3*	Mann–Whitney	U = 209.00	—	<0.001	$\|r\|$ = 1.000
F13	Days to flowering	*E3*	Kruskal–Wallis	H = 0.051	2	0.975	Rank ε^2 < 0.001
F13	Maturity status	*E3*	Pearson χ^2	1.584	2	0.453	Cramer's V = 0.168
F13	Days to maturity	*E3*	Kruskal–Wallis	H = 1.855	2	0.396	Rank ε^2 = 0.049
F13	Days to flowering	*E4*	Kruskal–Wallis	H = 11.93	2	0.003	Rank ε^2 = 0.217
F13	Maturity status	*E4*	Pearson χ^2	11.48	2	0.003	Cramer's V = 0.453
F13	Days to maturity	*E4*	Kruskal–Wallis	H = 11.41	2	0.003	Rank ε^2 = 0.300
F13	Days to flowering	*E7*-associated SSR marker class	Kruskal–Wallis	H = 5.959	2	0.051	Rank ε^2 = 0.108
F13	Maturity status	*E7*-associated SSR marker class	Pearson χ^2	15.72	2	<0.001	Cramer's V = 0.530
F13	Days to maturity	*E7*-associated SSR marker class	Kruskal–Wallis	H = 10.19	2	0.006	Rank ε^2 = 0.268
F13	Days to flowering	*E3 e4 e7* vs. *e3-fs e4 e7*	Mann–Whitney	U = 37.00	—	0.355	$\|r\|$ = 0.321
F13	Days to maturity	*E3 e4 e7* vs. *e3-fs e4 e7*	Mann–Whitney	U = 44.00	—	0.097	-
U4	Maturity status	*E1–E3* class	Pearson χ^2	2.182	3	0.536	Cramer's V = 0.302
U4	Days to flowering	*E1–E3* class	Kruskal–Wallis	H = 3.874	3	0.275	Rank ε^2 = 0.168
U4	Days to maturity	*E1–E3* class	Kruskal–Wallis	H = 4.379	3	0.223	Rank ε^2 = 0.209

B. Dunn's post hoc comparisons following significant Kruskal–Wallis tests

**Trait**	Factor	**Comparison**	**Dunn *z***	**Holm-adjusted *p***
Maturity	Population	F10 vs. F13	2.404	0.021
Maturity	Population	F10 vs. U4	4.324	<0.001
Maturity	Population	F13 vs. U4	2.556	0.021
Flowering	*E4*	*E4/E4* vs. *E4/e4*	−0.218	0.827
Flowering	*E4*	*E4/E4* vs. *e4/e4*	2.260	0.048
Flowering	*E4*	*E4/e4* vs. *e4/e4*	3.171	0.005
Maturity	*E4*	*E4/E4 vs. E4/e4*	0.627	0.531
Maturity	*E4*	*E4/E4 vs. e4/e4*	2.585	0.027
Maturity	*E4*	*E4/e4 vs. e4/e4*	2.616	0.027
Maturity	*E7*	*E7/E7vs. E7/e7*	−0.407	0.684
Maturity	*E7*	*E7/E7 vs. e7/e7*	1.334	0.364
Maturity	*E7*	*E7/e7vs. e7/e7*	3.112	0.006

https://doi.org/10.3390/life16101663
